# The Sutures for Vesico-Vaginal Fistula Inserted by Means of Canula-needle, with Report of a Case

**Published:** 1869-05

**Authors:** Paul F. Eve

**Affiliations:** Nashville


### The Sutures for Vesico-Vaginal Fistula inserted by means of Canula-needle, with Report of a Case.

BY PROF. PAUL F. EVE, OF NASHVILLE.

In 1860, I utterly failed to benefit a patient laboring under that most distressing affection in the female, *vesico-vaginal fistula*, in which a clamp suture was tested. There is little doubt now but that this case could have been relieved by the recent improvements in gynecology. Still I have never been satisfied with the practice pursued in introducing the sutures for closing the fistulous opening, after its edges are prepared for reunion. The short, curved needle, insecurely held and imperfectly directed by long forceps, armed with a long, double silk thread, having attached to it a long silver wire, also doubled, the whole of which is dragged through the entire tract made in the soft parts, with the traction on the cord at right angle to its proper course, is surely difficult, cumbrous, painful, tedious, unscientific, and now unnecessary. Frequently half an hour is consumed in this part of the operation, while the patient is in a constrained and exceedingly unpleasant position. By the canula-needle the ligatures may be applied at any desired point in a few minutes. In witnessing an operation for this sloughing of the vesico-vaginal septum, to which I was kindly invited in the Sisters' Hospital in St. Louis last fall, and, impressed with the difficulty and delay in applying the sutures, the hollow needle was proposed, and fortunately an opportunity soon occurred for proving it.

On the fifteenth of November last, I was called to a case of vesico-vaginal fistula in a young lady, who, during her first delivery, a week or two previously, had had the bladder neglected for many hours, which resulted in a transverse fissure of about an inch and a half by three-quarters of an inch, and situated about an inch and a half from the orifice of the urethra. The sixteenth of January, 1869, assisted by Dr. Shumard, professor of obstetrics, etc., in the Missouri Medical College, and Drs. Poindexter, Prewitt, Moore and Maughs, the patient was placed in the position now recommended by Dr. Sims, and accepted by the profession; the anterior lip of the fistula was freely excised by a knife and the posterior one with scissors, then with the canula-needle, such as I have been using for years, carrying a very fine silver wire, about nine sutures were introduced in some ten minutes. Tieman's improvement, as well as Dr. Bryant's, formerly of St. Louis, now of Lexington, Ky., each consisting of a longer canula, with forceps to hold and assist in pushing forward the wire, were both tried, but found to bend in thrusting it through the parts, and, moreover, permitted the metallic ligature to kink. The simple canula, was admitted by all present to be the best instrument and gave entire satisfaction in planting the ligatures. I prefer, too, the ordinary knot, for one can see the line of union and regulate the pressure in tightening the stitch. The suture-adjuster obscures this, nor can we tell how many turns to give the forceps in twisting the wire. After one knot, the adjuster may be used.

On the tenth day, after the usual treatment of such cases, which, in this instance, was carried out faithfully, the sutures were all removed, except two, and these on the twelfth, when union of the most satisfactory character was found existing.—*Richmond and Louisville Medical Journal.*

